# Nonlinear Earthquake Analysis of Reinforced Concrete Frames with Fiber and Bernoulli-Euler Beam-Column Element

**DOI:** 10.1155/2014/905963

**Published:** 2014-01-22

**Authors:** Muhammet Karaton

**Affiliations:** Civil Engineering Department, Engineering Faculty, Fırat University, 23119 Elazig, Turkey

## Abstract

A beam-column element based on the Euler-Bernoulli beam theory is researched for nonlinear dynamic analysis of reinforced concrete (RC) structural element. Stiffness matrix of this element is obtained by using rigidity method. A solution technique that included nonlinear dynamic substructure procedure is developed for dynamic analyses of RC frames. A predicted-corrected form of the Bossak-**α** method is applied for dynamic integration scheme. A comparison of experimental data of a RC column element with numerical results, obtained from proposed solution technique, is studied for verification the numerical solutions. Furthermore, nonlinear cyclic analysis results of a portal reinforced concrete frame are achieved for comparing the proposed solution technique with Fibre element, based on flexibility method. However, seismic damage analyses of an 8-story RC frame structure with soft-story are investigated for cases of lumped/distributed mass and load. Damage region, propagation, and intensities according to both approaches are researched.

## 1. Introduction

The realistic modeling of the nonlinear static or dynamic behavior of RC structures is a more sophisticated problem due to the inelastic behavior of concrete, plasticity of reinforcement, interface debonding of these materials, and so on. In the last thirty years, researchers have made more effort for the numerical modeling of RC structures and most of the state-of-the-art on this problem deals with two main approaches: lumped plasticity modeling [1–3] and distributed-inelasticity modeling (i.e., the so-called fibre beam-column elements, FBCE) [4–8]. In the first approach, nonlinear springs based on moment-rotation and force-displacement curves are used and nonlinear volume is assumed to be lumped in the specific location of the element. Mohr et al. [1] were used a series of polynomial shape functions for strain distributions of the vertical and shear on cross-section. Li et al. [2] was proposed a simplified lumped hinge for determination of failure mode of statically indeterminate structure. Reshotkina and Lau [3] was used a concentrated plasticity approach for axial-flexure-shear interactions on the inelastic behavior of reinforced concrete members under the seismic loads. In the second approach, the nonlinear behaviors of concrete and reinforcement materials are separately calculated. This method divided into two as approaches was based on flexibility and rigidity. Taucer et al. [9] were developed a flexibility method for nonlinear dynamic analysis of reinforced concrete element. This method based on Bernoulli-Euler hypothesis included biaxial bending and axial force conditions. Nonlinear behavior of concrete and steel were computed by using uniaxial stress-strain relationships. Force-displacement interpolation functions are used for the obtaining of element flexibility matrix. Element stiffness matrix is achieved by inverse of flexibility matrix. Furthermore, Ceresa et al. [10] were developed a beam-column element based on flexibility matrix by using to the Timoshenko beam theory under cyclic loading. Damages in the element are taken into account with coaxial rotating crack model. Lu et al. [4] were used multilayered shell element and fibre beam-column element based on flexibility method for modeling of the different frame and frame with shear wall. However, an important research report about methods based on rigidity was performed by Kawano et al. [6]. In the report, nonlinear analyses of reinforced concrete space frames with multistory and multibay are obtained under the earthquake loads. Cubic Hermitian polynomials for transversal displacements and the linear shape functions for axial displacements are used for obtaining of stiffness matrix. However, the Gauss-Lobatto integration rule was used for obtaining element mass and stiffness matrices. Element damage location was simply presented and element was not divided into subelement called as “segment.” Some calculation problems in heavily damage location was shown and they are reported to arise from flexural deformation. Furthermore, strain rate effects in response to reinforced concrete frames was investigated by Iribarren [7]. Used formulations are based on rigidity method and a strain rate dependent material formulation is developed for both the concrete and steel constitutive response. Brum [8] was obtained nonlinear dynamic analyses of frame and masonry structures by using a method which was based on stiffness matrix. In the research, a uniaxial constitutive model for concrete and masonry is proposed for compression and tension regions of the materials under the cyclic loadings.

In this study, a beam-column element based on the Euler-Bernoulli beam theory is presented for the fiber RC element. Element stiffness matrices are obtained by using rigidity method. The beam or column element is divided into subelements called “segment” [11]. Furthermore, the internal freedoms of this segment are dynamically condensed to external freedoms at the end of the element. Thus, nonlinear dynamic analysis of high RC building can be obtained within a short time. This procedure requires that nested loops of the element and structure are obtained at the same time (the so-called nonlinear dynamic substructure). However, this condensation procedure is not used in the modeling of the fibre element approach (FEA) of the RC element. In addition, uniform or trapezoidal loads of the segment are assumed to be zero or condensed to the external freedoms at the end of the element in the FEA [11]. This case is not preferred due to the required redistribution of loading. However, in this study, external loading of the segment is taken into account by considering the damage occurred in the element.

The present research is organized as follows: (i) presenting of fiber Bernoulli-Euler beam-column element based on rigidity method, (ii) developing of nonlinear dynamic substructure technique of RC frames, (iii) verification of the constitutive model with respect to experimental result of a column structural element and dynamic analysis results of fibre RC element of a portal frame, (iv) obtaining of seismic damage analyses of an 8-story RC frame with soft-story for distributed/lumped mass and load case, and (v) results.

## 2. Fiber and Bernoulli-Euler Approach (FBEA) for Reinforced Concrete Beam Column Element

In this section, theory of Fiber and Bernoulli-Euler element which is based on stiffness matrix is firstly presented for reinforced concrete section. Elements are divided into subelements, called segment, and they are assumed as substructures of element. Furthermore, cross-section of the each segment is also subdivided into a number of fibers/layers. In the next section, nonlinear dynamic substructures method is also mentioned to be applied to freedoms in element ends.

### 2.1. Obtaining Segment Stiffness and Mass Matrices

**Figure 1:**
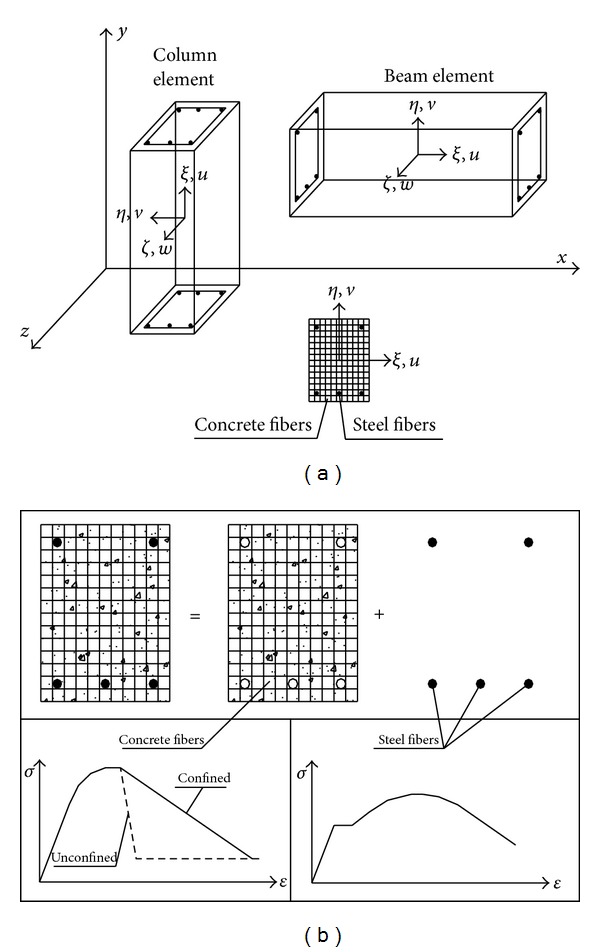
Concrete and steel fibers and global and local axes for FBEA.

The strain distribution on a cross-section of beam is assumed to be uniform due to axial forces and linear due to only bending according to the Bernoulli-Euler approach. Furthermore, if a plane section before bending is plane after bending and if the strain is assumed to be small and shear stresses are omitted, the strain of a point on a cross-section in the axial direction can be written as

(1) $$\begin{array}{c} \varepsilon_{xx}=\frac{du}{dx}-\frac{d^{2}v}{dx^{2}}y, \end{array}$$

where *u* and *v* are the displacement of the axial and vertical directions of element, respectively (Figure 1). However, if cross-section of element is divided into fibers/layers, this equation for each fiber/layer in local axis can be rewritten as,

(2) $$\begin{array}{c} \varepsilon_{\xi ,n}=\left\{\begin{array}{cc} 1 & -\eta_{n}^{\prime } \end{array}\right\}\left\{\begin{array}{c} \frac{du}{d\xi }\\ \frac{d^{2}v}{d\xi^{2}} \end{array}\right\}, \end{array}$$

where *dε*_*ξ*,*n*_ is incremental axial strain of a fiber/layer on a segment in *ξ* local axis direction. Therefore, if the cubic Hermitian polynomials for transversal displacements and the linear shape functions for axial displacements are used [12], (2) can be written as

(3) $$\begin{array}{c} \varepsilon_{\xi ,n}=\left\{\begin{array}{cc} 1 & -\eta_{n}^{\prime } \end{array}\right\}\left\{\begin{array}{c} \frac{d\left[N\left(\xi \right)\right]}{d\xi }\\ \frac{d^{2}\left[N\left(\xi \right)\right]}{d\xi^{2}} \end{array}\right\}\left\{q\right\}=\left\{\begin{array}{cc} 1 & -\eta_{n}^{\prime } \end{array}\right\}\left[B\right]\left\{q\right\}, \end{array}$$

where {*q*} is displacement vector, including displacement and rotation of a segment. [*B*] is strain-displacement matrix. Thus, incremental stress in each fiber/layer can obtained as

(4) $$\begin{array}{c} d\sigma_{\xi ,n}=E_{T,n}d\varepsilon_{\xi ,n}=E_{T,n}\left\{\begin{array}{cc} 1 & -\eta_{n}^{\prime } \end{array}\right\}\left[B\right]\left\{q\right\}, \end{array}$$

where *E*_*T*,*n*_, determined by using uniaxial stress-strain relationship of using materials, is tangent elasticity modulus of each fiber. Therefore, total strain energy of a segment subelement is obtained as

(5) Π=12∫LSegσTεASegdx=12∫−11{q}T[B]T[{1−ηn′}ET,nA{1−ηn′}][B]{q}dξ=  12∫−11{q}T[B]T×[∑n=1NET,nAn−∑n=1NET,nAnηn′−∑n=1NET,nAnηn′∑n=1NET,nAn(ηn′)2][B]{q}dξ,

where *L*_Seg_ and *A*_Seg_ are expressed to be length and cross-section area of segment, respectively. *A*_*n*_ is also area of a fiber/layer. Thus, element stiffness matrix can be obtained by using minimum potential energy principle. Stiffness matrix of a segment can be written as

(6) [KSeg] =12∫−11[B]T[∑n=1NET,nAn−∑n=1NET,nAnηn′−∑n=1NET,nAnηn′∑n=1NET,nAn(ηn′)2][B]dξ.

The segment stiffness matrix is obtained by using areas, coordinates, and tangent elasticity modulus of fiber/layer for reinforced concrete sections. In this study, linear superposition rule is used for different material properties of the fiber/layer in the section (Figure 1). Furthermore, a fiber concrete or reinforced bar on the cross-section may be cracked or damaged due to external loading. For this reason, a relationship between the damaged and undamaged cases must be obtained for the solutions. Thus, damage in each concrete/reinforcement fiber can be written as [13]

(7) $$\begin{array}{c} d_{n}=1-\frac{E_{T,n}}{E_{O,n}}, \end{array}$$

where *E*_*O*,*n*_ and *E*_*T*,*n*_ are undamaged and damaged/tangent elasticity module of the *n*th fiber, respectively. *d*_*n*_, damage intensities are obtained separately under tensile and compressive stress for each fiber.

However, total kinetic energy of particle velocities on the cross-section of an element throughout its neutral axis can be written as

(8) $$\begin{array}{c} T=\frac{1}{2}\int_{\Omega }v_{\xi }\rho v_{\xi }d\Omega , \end{array}$$

where *ρ* is mass density and *v*_*ξ*_ is particle velocity. If fiber/layer elements are used for (8), the mass matrices of an element on the local axis are obtained as

(9) $$\begin{array}{c} \left[M_{\text{Seg}}\right]=\int_{-1}^{1}\sum_{n=1}^{nfib}\left({\left[N\left(\xi \right)\right]}^{T}\rho_{n}A_{n}\left[N\left(\xi \right)\right]\right)d\xi , \end{array}$$

where [*N*(*ξ*)] is the element shape functions matrix on the local axis and *ρ*_*n*_ is also mass density of the *n*th fiber. Stiffness and mass matrices of a segment, obtained by local axis, are transformed to global axes by using transformation matrix.

### 2.2. Obtaining with Nonlinear Dynamic Substructures Technique of Element Stiffness and Mass Matrices

**Figure 2:**
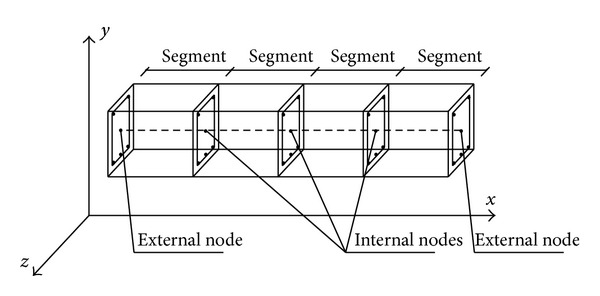
Segments and external and internal nodes for FBEA.

In FBEA, cubic Hermitian shape functions for rotation and shear strain and linear shape functions for the axial deformation are used, respectively. The Gauss-Lobatto integration rule is generally used for obtaining element mass and stiffness matrices along with *ξ* local axis [6–8]. However, substructure procedures are generally preferred for solutions of high buildings. However, this technique in the previous study is not used due to numerical difficultly. Element stiffness/flexibility matrix is obtained by using cross-section properties on the integration points. Therefore, effects of shape functions on the solution are very important. In this study, an element is divided into subelements which are called “segment” to remove disadvantage effects of the shape functions. The freedoms of segment are condensed to the end freedoms at the ends of the element. Thus, if freedoms at ends and internal regions of element are called as external and internal freedoms, respectively (Figure 2), these external and internal freedoms for stiffness matrices can be written as,

(10a) $$\begin{array}{c} \left[K_{EE}\right]\left\{u_{E}\right\}+\left[K_{EI}\right]\left\{u_{I}\right\}=\left\{F_{E}\right\}, \end{array}$$

(10b) $$\begin{array}{c} \left[K_{IE}\right]\left\{u_{E}\right\}+\left[K_{II}\right]\left\{u_{I}\right\}=\left\{F_{I}\right\}, \end{array}$$

where [*K*_*EE*_] and [*K*_*II*_] are stiffness matrices which include external and internal freedoms, respectively [11]. {*u*_*E*_} and {*u*_*I*_} are the displacement vectors referred by these freedom; {*F*_*E*_} and {*F*_*I*_} are also the external loads of these freedoms. When applying the substructure procedure to (10a) and (10b), the external load and stiffness matrices of a frame element can be rewritten as

(11a) $$\begin{array}{c} \left[K_{EE,F}\right]\left\{u_{E}\right\}=\left\{F_{E,F}\right\}, \end{array}$$

(11b) $$\begin{array}{c} \left[K_{EE,F}\right]=\left[K_{EE}\right]-\left[K_{EI}\right]{\left[K_{II}\right]}^{-1}\left[K_{IE}\right], \end{array}$$

(11c) $$\begin{array}{c} \left\{F_{E,F}\right\}=\left\{F_{E}\right\}-\left[K_{EI}\right]{\left[K_{II}\right]}^{-1}\left\{F_{I}\right\}. \end{array}$$

However, if the Rayleigh method may be used to obtain element damping matrices, this matrix is defined as

(12) [[CEE][CEI][CIE][CII]] =αdam[[MEE][MEI][MIE][MII]]+βdam[[KEE][KEI][KIE][KII]],

where *α*_dam_ and *β*_dam_ are Rayleigh damping coefficients with respect to mass and stiffness matrices, respectively [14]. However, if substructure procedure in (11a), (11b), and (11c) is applied to mass and damping matrices [15],

(13a) [MEE,F]=[MEE]−[KEI][KII]−1[MIE]−[MEI][KII]−1[KIE]+[KEI][KII]−1[MII][KII]−1[KIE],

(13b) [CEE,F]=[CEE]−[KEI][KII]−1[CIE]−[CEI][KII]−1[KIE]+[KEI][KII]−1[CII][KII]−1[KIE],

mass and damping matrices can be obtained for the external freedom. Global stiffness, damping, and mass matrices may be achieved by using the stiffness and mass matrices belonging to external freedom. Thus, global stiffness, mass matrices, and external load vector can be calculated as

(14) [KS]=∑k=1nelem[KEE,F],  [CS]=∑k=1nelem[CEE,F],[MS]=∑k=1nelem[MEE,F],  {FS}=∑k=1nelem{FE,F}.

Therefore, the dynamic equilibrium equations of the structure may be given as

(15) [MS]{aE}t+Δt+[CS]{vE}t+Δt   +[KS]{uE}t+Δt={FS,gr}t+Δt+{FS,stat},

where subscripts gr and stat indicate that the quantity is related to ground acceleration and static loads.

### 2.3. Bossak-*α* Form of the Equation of Motion

The equation of motion for the RC frame is given in (15). In this study, the Bossak-*α* integration method, presented by Wood et al. [16], is used for the solution of the equation in the time domain. Integration scheme of the method retains the Newmark method. Furthermore, the Bossak-*α* integration method is required to be modified to (15) in the time domain as follows:

(16) (1−αB)[MS]{aS}t+Δt+αB[MS]{aS}t   +[CS]{vS}t+Δt+{FS,res}t+Δt  =(1−αB){FS,gr}t+Δt+αB{FS,gr}t+{FS,stat},

where *α*_*B*_ is the Bossak parameter, used for controlling the numerical dissipation. The Bossak parameter should be chosen as in (17) for unconditional stability and second-order accuracy:

(17) $$\begin{array}{c} \alpha_{B}\leq \frac{1}{2};\;\;\beta =\frac{1}{4}{\left(1-\alpha_{B}\right)}^{2};\;\;\gamma =\frac{1}{2}-\alpha_{B}. \end{array}$$

In this study, *α*_*B*_ is selected as −0.10. To solve the nonlinear dynamic equation of motion for the RC frame, the Newton-Raphson method is used in conjunction with the predictor-corrector technique. The Bossak-*α* time integration algorithm is given by Wood et al. [16]. Predicted displacement and velocity vectors for the time step *t* + Δ*t* are obtained by using displacement and velocity vectors at the time step *t* which is known. Thus, they can be calculated as

(18a) $$\begin{array}{c} {\left\{{\tilde{u}}_{S}\right\}}_{t+\Delta t}={\left\{u_{S}\right\}}_{t}+\Delta t{\left\{v_{S}\right\}}_{t}+\frac{1}{2}\Delta t^{2}\left(1-2\beta \right){\left\{a_{S}\right\}}_{t+\Delta t}, \end{array}$$

(18b) $$\begin{array}{c} {\left\{{\tilde{v}}_{S}\right\}}_{t+\Delta t}={\left\{v_{S}\right\}}_{t}+\Delta t\left(1-\gamma \right){\left\{a_{S}\right\}}_{t}, \end{array}$$

where *β* and *γ* are Newmark's coefficients. The displacement and velocity vectors [17] of the RC frame can be written in terms of the predicted vectors shown in (18a) and (18b). The vectors may be defined as

(19a) $$\begin{array}{c} {\left\{u_{S}\right\}}_{t+\Delta t}={\left\{{\tilde{u}}_{S}\right\}}_{t+\Delta t}+\beta \Delta t^{2}{\left\{a_{S}\right\}}_{t+\Delta t}, \end{array}$$

(19b) $$\begin{array}{c} {\left\{v_{S}\right\}}_{t+\Delta t}={\left\{{\tilde{v}}_{S}\right\}}_{t+\Delta t}+\Delta t\gamma {\left\{a_{S}\right\}}_{t+\Delta t}. \end{array}$$

These relations can be substituted into (16) and a time marching algorithm can be applied to this equation as given in the Appendix.

## 3. Numerical Applications

### 3.1. Comparison of Experimental and Numerical Analysis of a RC Column

**Figure 3:**
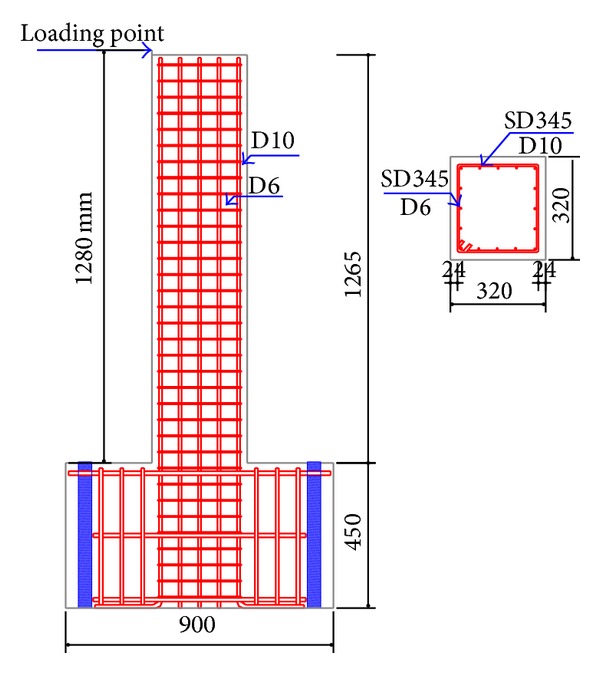
Experimental setup and geometrical properties of RC column.

**Figure 4:**
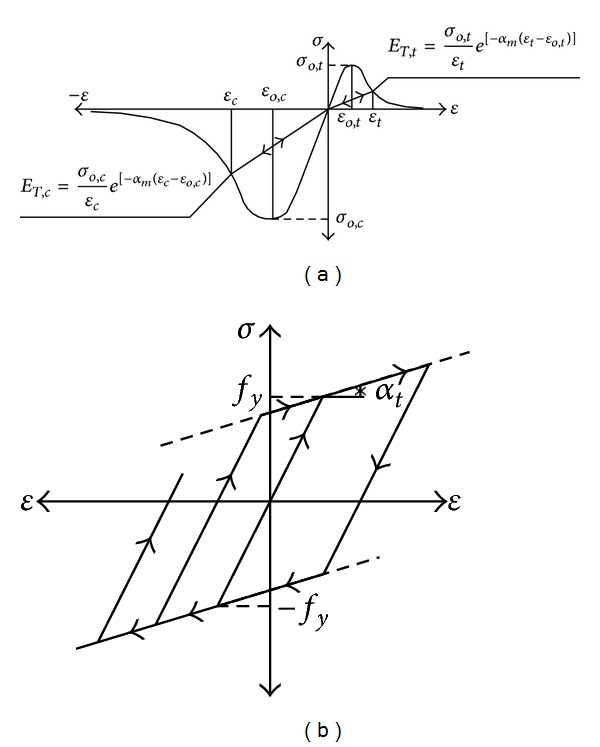
Stress-strain relationships of (a) concrete and (b) steel.

In this section, experimental cyclic test results of a RC column with numerical solutions obtained from proposed solution method are compared. The experimental test result includes response under uniaxial static and lateral cyclic forces. Loading and material properties of the experimental study are given by Takahashi [18]. The experimental setup is shown in Figure 3. Exponential decreasing functions for the softening region of tensile and compressive strengths of the concrete for the constitutive model of FBEA are used. These functions are shown in Figure 4(a). The bilinear kinematic hardening rule is used for nonlinear behavior of the steel for the two approaches (Figure 4(b)). Static loads are converted to masses which are condensed to the element ends for all solutions and displacement values obtained due to the loads being considered as the initial condition. Acceleration data, a sinus wave shape, and time varying are used for all dynamic solutions. This dynamic load is applied to the horizontal direction at 1280 mm height of RC column. Tangent stiffness matrix is used for the solution and damping matrix is also assumed to be proportional to stiffness matrix.

**Figure 5:**
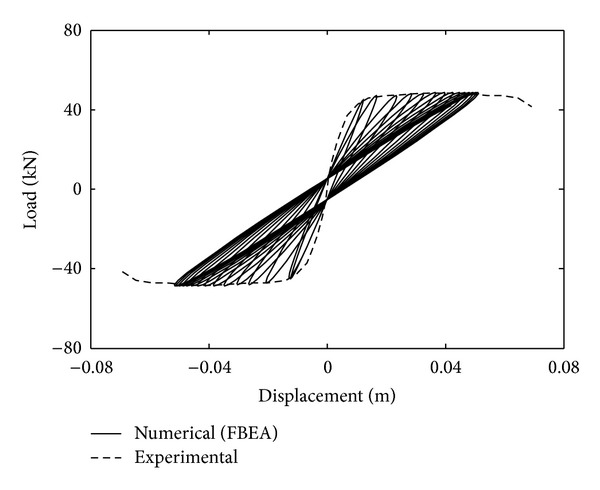
Comparison of experimental and numerical responses of RC column.

Load-displacement curves of experimental and numerical results are given in Figure 5. Maximum and minimum values of cyclic displacement obtained from numerical results are approximately between −0.055 and 0.055 m. Numerical response of RC element which is obtained with FBEA is shown as similar to envelope curve of experimental results. All values of the horizontal displacements are on the envelope curve of the experimental result. It is said that this solution technique and material models of concrete and steel can be used for the solution of the RC structural element under the dynamic loading.

### 3.2. Nonlinear Dynamic Analyses of a Portal RC Frame Structure

**Figure 6:**
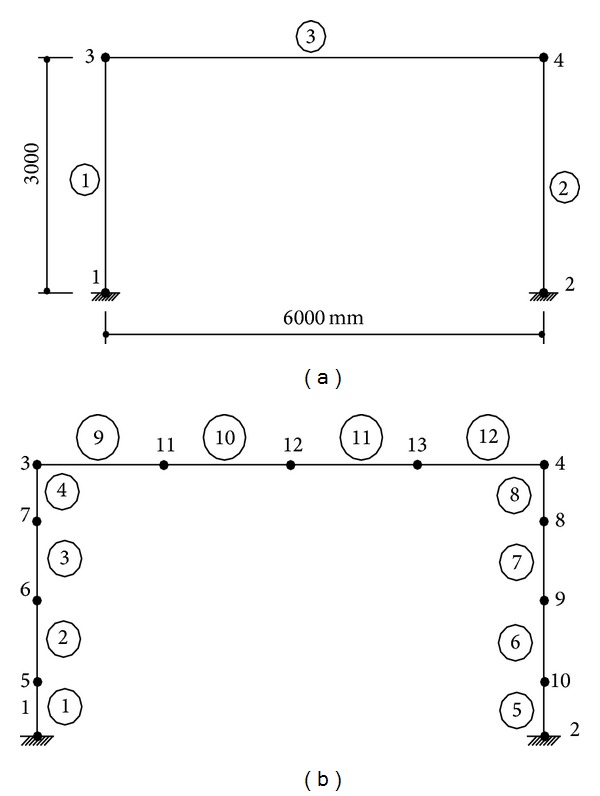
Finite element mesh for (a) FEBA and (b) FEA.

In this section, nonlinear dynamic analyses of a portal RC frame are obtained for the comparing of proposed solution technique with model based on flexibility. Seismo-Structure program [19] is used for the flexibility based solutions (with fibre element method). Finite element meshes are shown in Figure 6 for both approaches. Finite element mesh of proposed method is divided into segment for comparing with analysis results of Seismo-Structure Program. ACI 318-02 [20] code is used for the material properties of concrete and steel. Cross-section and material properties of the beam and column of selected portal RC frame are given in Table 1. The tensile softening region in the fibre element method is not taken into account while the compressive behavior of the concrete is used. In Seismo-Structure program, if the tensile stresses obtained from the solutions reach the tensile strength of the concrete, tensile strength suddenly drops to zero. In this case, a sudden collapse of the structure occurs.

**Figure 7:**
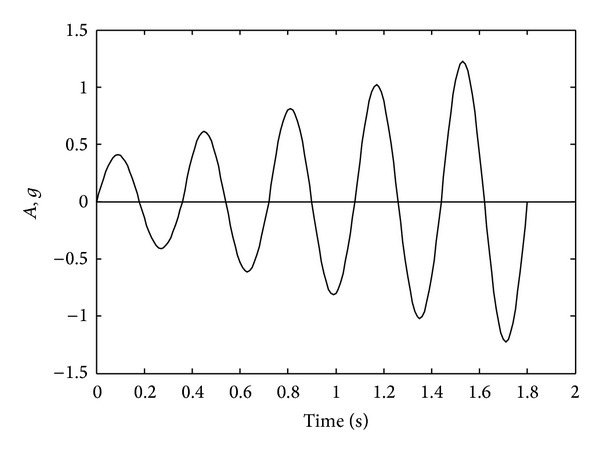
Time history graphs of sinus acceleration.

In this study, exponential decreasing functions in the softening region of tensile and compressive strengths of the concrete for constitutive model of FBEA are used. These functions are shown in Figure 4(a). The bilinear kinematic hardening rule is used for nonlinear behavior of the steel for the two approaches (Figure 4(b)). Static loads are converted to masses which are condensed to the element ends for all solutions and displacement values obtained due to the loads being considered as the initial condition. Acceleration data and a sinus wave shape are used for all dynamic solutions (Figure 7). This dynamic load is applied to the horizontal direction.

**Figure 8:**
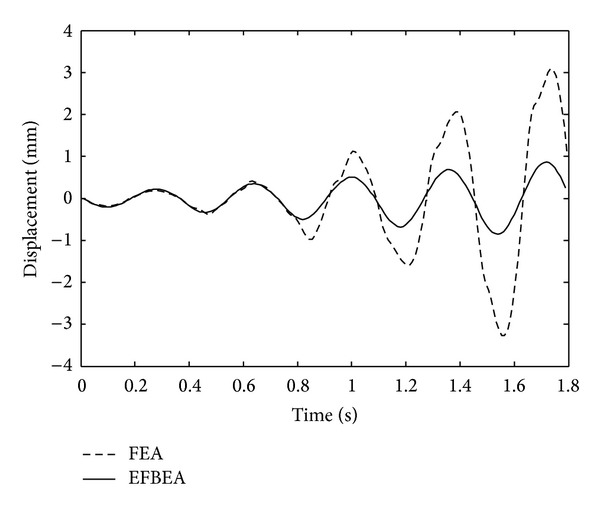
Displacement time history graphs of node 3 for FEA and FBEA.

Displacement time history graphs of node 3 obtained from FBEA and FEA are shown in Figure 8. Displacement time amplitude values are approximately similar until time of 0.75 sec and, after this time, displacement amplitude values obtained from the FEA are bigger than those obtained from the FBEA. This case arises from not taking into account the concrete tensile softening region for the fibre element method. However, failure of structure is not seen for two approaches in all times.

**Figure 9:**
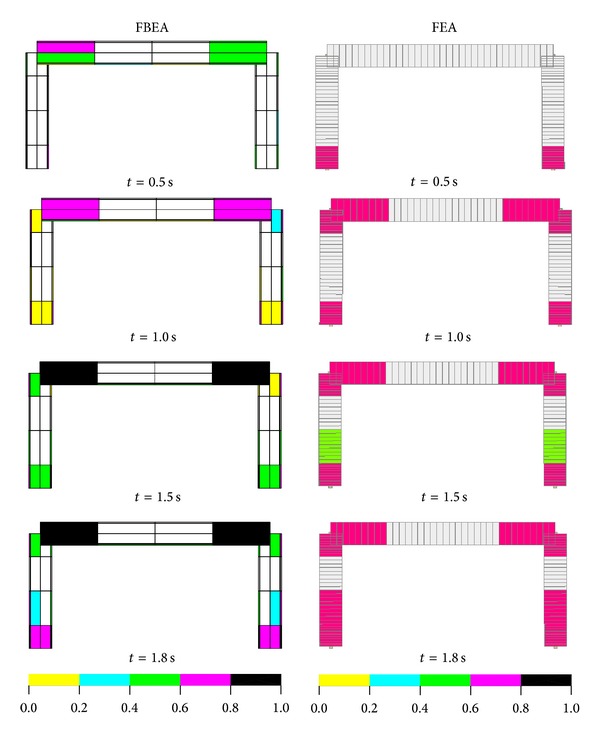
Accumulated tensile damage zones of the portal frame.

Accumulated tensile damage cases obtained for both approaches are given in Figure 9. The first damage zones are obtained at the whole of end regions of the beams and at outside surfaces of bottom regions of the columns for the FBEA. Damages are shown at the whole cross-section of bottom regions of the columns for the FEA. Furthermore, obtained damage zone regions in the beam and column for the FBEA and FEA are similar at *t* = 1.0 sec. However, some differences between both approaches are seen from the point of intensities of the damage at this time. However, damage intensities at end regions of the beam according to FBEA are extended and damage regions are propagated to middle region of the beam at the *t* = 1.5 sec. Damage intensities at end regions of the column are also extended and damage regions are propagated to middle region from upper region of the column at the *t* = 1.5 sec. Additionally, damage intensities at end regions of the beam according to FEA are not extended at this time. Damage intensities at end regions of the column are also extended and damage regions are propagated to middle region from upper region of the column; after this time, damage zones are extended but propagation of damages are not seen according to both approaches at the *t* = 1.8 sec.

### 3.3. Seismic Damage Analyses of an 8-Story RC Frame Structure with Soft-Story

**Figure 10:**
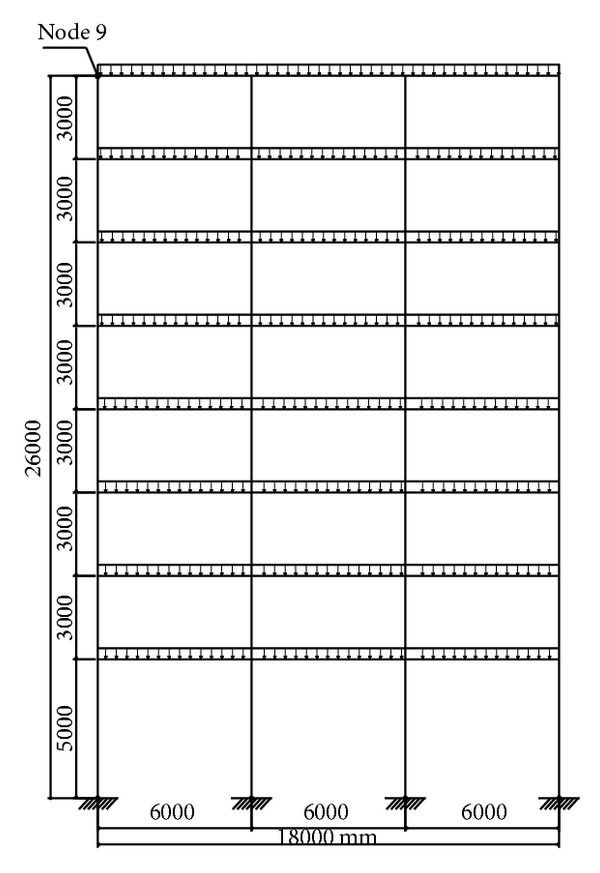
Finite element mesh of 8-story RC frame with soft-story.

**Figure 11:**
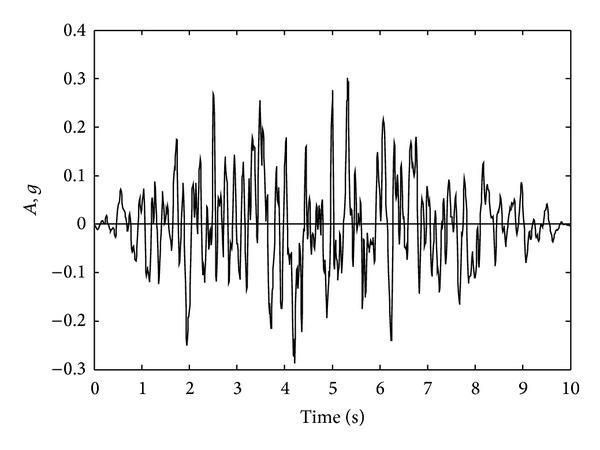
Time history graphs of synthetic acceleration.

In this numerical application, nonlinear dynamic analyses of an 8-story RC frame structure with soft-story are investigated for cases of lumped/distributed mass and load. ACI 318-02 [20] code is used for the material properties of concrete and steel. Cross-section and material properties of the beam and column of selected RC frame are given in Table 2. Finite element mesh and gravity loading case are shown in Figure 10. Uniaxial stress-stain relationships of the concrete and steel are seen in Figures 4(a) and 4(b), respectively. Static loads and displacements are considered as initial conditions in all solutions. Spectrum acceleration curve, given by Z1 type soil in the Turkish Regulation Code on Building in Disaster Areas [20], is selected as the target spectrum curve. Synthetic earthquake acceleration data for maximum amplitude, 0.3 g, are produced. This synthetic acceleration-time graph is shown in Figure 11 and it is affected on the horizontal direction of the RC frame structure. Tangent stiffness matrix is used for the all solution and damping matrix is also assumed to be proportional to stiffness matrix.

**Figure 12:**
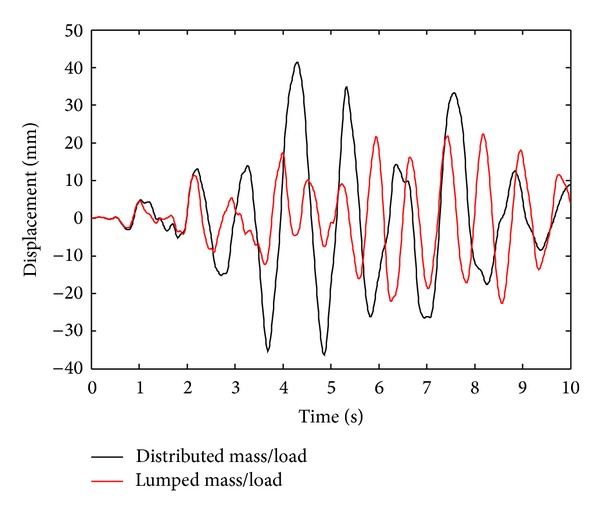
Horizontal displacement time history graphs of node 9 for lumped/distributed mass and load cases.

Displacement time history graphs of node 9 obtained from nonlinear dynamic analyses results for lumped/distributed mass and load cases are shown in Figure 12. Displacement amplitude values are approximately similar until time of 1.08 sec and, after this time, displacement amplitude values obtained from the distributed mass and load case are bigger than those obtained from the lumped case. Absolute maximum horizontal displacements for the lumped and distributed cases are obtained as 22.6 and 41.4 mm, respectively. Thus, absolute maximum displacement value according to distributed case is approximately 83% of ratio bigger than that of lumped case. This result arises from the redistribution of loading in the internal regions of the elements for the distributed approach. However, the redistribution of loading in these internal regions is only obtained at the end regions of elements for the lumped approach. The redistribution procedure for loading in these internal regions requires more iterative steps for a distributed approach. However, numerical dissipation is not shown for both solutions and Newton-Raphson procedures for all element solutions which are converged for the two approaches. The Bossak-*α* dynamic integration algorithm is applied successfully to the nonlinear dynamic solutions.

**Figure 13:**
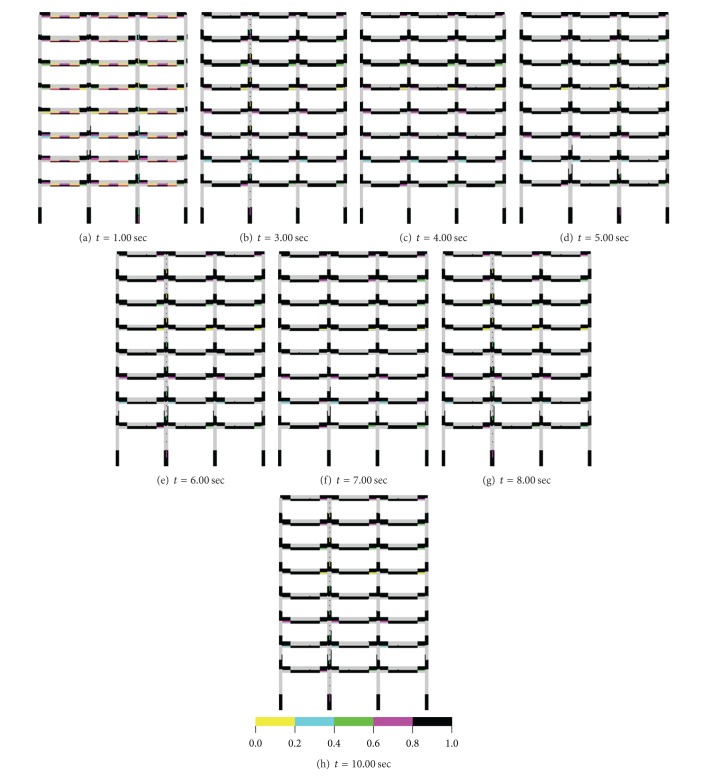
Accumulated damage zones in building for distributed mass/loading case.

**Figure 14:**
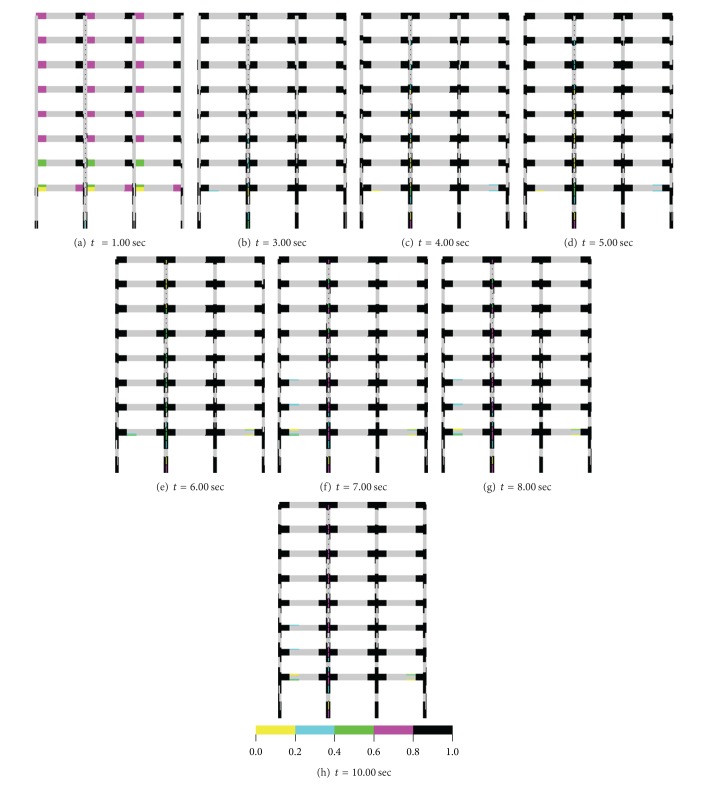
Accumulated damage zones in building for lumped mass/loading case.

Accumulated tensile damage regions obtained for both mass/load case are given in Figures 13 and 14. Damage zones in the lumped case are obtained at the end of the beam of all floors and at the whole of the beam cross-section for the time = 1.00 sec. Damage zones occurred at upper parts of the beams at the beam-column join region and at lower parts of the mid region of the beams until this time for the distributed case. Furthermore, damage zones seen at lower parts of first-floor columns occurred for both approaches, but intensities and regions of these damages according to distributed approach are bigger than those of lumped mass/load case. The damage intensities are some increased and damage propagations are remained at the same regions for both approaches until *t* = 3.00 sec; after this time, damage intensities according to two approaches are increased at between *t* = 3.00 and 8.00 sec, and no change for the damage zones is obtained. However, increases in the damage intensities achieved a minimal level between *t* = 8.00 and 10.00 sec. It is said that the damage zones obtained according to the distributed/lumped case had important differences.

## 4. Conclusions

In this study, a beam-column element based on the Euler-Bernoulli beam theory is researched for nonlinear dynamic analysis of reinforced concrete (RC) structural element. Stiffness matrix of this element is obtained by using rigidity method. A solution technique that included nonlinear dynamic substructure procedure is developed for dynamic analyses of RC frames. A predicted-corrected form of the Bossak-*α* method is applied to dynamic integration scheme. A comparison of experimental data of a RC column element with numerical results, obtained from proposed solution technique, is studied for verification of the numerical solutions. Furthermore, nonlinear cyclic analysis results of a portal reinforced concrete frame are achieved for comparing of the proposed solution technique with fibre element, based on flexibility method. However, seismic damage analyses of an 8-story RC frame structure with soft-story are investigated for cases of lumped/distributed mass and load. Damage region, propagation, and intensities according to both approaches are researched. Results obtained from this study are presented to be itemized as follow.

- Constitutive model based on rigidity method is used in obtaining the stiffness matrix. The beam or column element is divided into a subelement called the segment. The internal freedoms of this segment are dynamically condensed to the external freedoms at the ends of the element. FBEA requires less freedom than the FEA due to the nonlinear dynamic substructure. Thus, nonlinear dynamic analysis of high RC building can be obtained within short times. This condensation procedure in previous study is not used for the modeling of the FBEA of RC element.
- Numerical response of RC element which obtained with FBEA is similar to envelope curve of experimental results. All values of the horizontal displacements are on the envelope curve of the experimental result. It is said that this solution technique and material models of concrete and steel can be used for the solution of the RC structural element under the dynamic loading.
- Accumulated tensile damage cases obtained for both FBEA and FEA approaches are similar, but some differents for displacement values obtained according to both methods are seen. These differents are to be small for little values of tensile damage intensities and propagation regions of damage and are to be big depending on increasing of the damage intensities and propagation regions of damage. However, tensile damage regions are obtained in detail according to the FBEA.
- When the results obtained from lumped and distributed approaches are investigated, absolute maximum displacement values for distributed approach are seen to be bigger than those for lumped approach. This case arises from the redistribution of loads in the internal regions of the elements for the distributed approach. However, the redistribution of loads in these internal regions is not used for the lumped approach. The redistribution procedure for loading in these internal regions requires more iterative steps.
- Obtained damage zones for the lumped mass/load case are seen at the end of the beam of all floors and at the whole of the beam cross-section, but obtained damage regions for the distributed mass/load case showed up at upper parts of the beams at the beam-column join region and at lower parts of the mid region of the beams. Furthermore, additional damage zones at lower parts of first-floor columns occurred for both approaches, but intensities and regions of these damages according to distributed approach are bigger than those of lumped approach.
- However, numerical dissipation is not shown for all solutions and Newton-Raphson procedures for all element solutions are converged for the two approaches. The Bossak-*α* time integration algorithm and proposed solution technique are successfully applied to the nonlinear dynamic solutions.

## Figures and Tables

**Table 1:** Material and cross-section properties of the portal frame example.

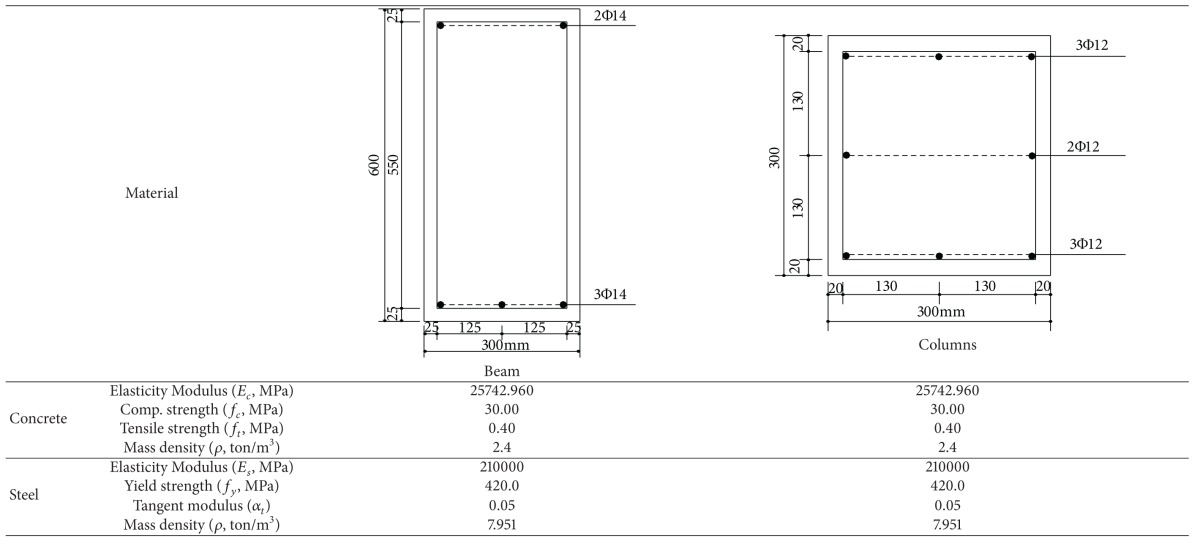

**Table 2:** Material and cross-section properties of 8-story RC frame.

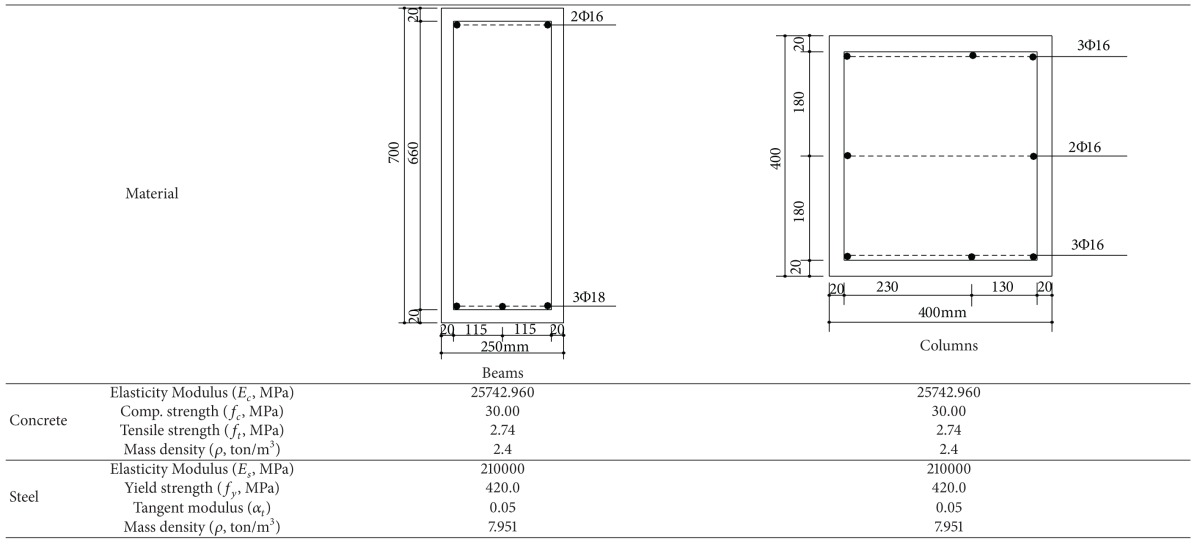

## Appendix

### Time Marching Algorithm

The algorithm applied to (16) is as follows.

(1) Compute integration parameters
  (A.1) $$\begin{array}{c} A_{1}=\frac{1}{\beta \Delta t^{2}};\;\;A_{2}=\frac{\gamma }{\beta \Delta t}. \end{array}$$
(2) {*u*_*S*_}_*t*+Δ*t*_, {*v*_*S*_}_*t*+Δ*t*_, and {*a*_*S*_}_*t*+Δ*t*_ are known; set global iteration counter, *i* = 1.
(3) Predict response at *t* + Δ*t*,
  (A.2a) $$\begin{array}{c} {\left\{u_{S}\right\}}_{t+\Delta t}^{i}={\left\{{\tilde{u}}_{S}\right\}}_{t+\Delta t}, \end{array}$$
  (A.2b) $$\begin{array}{c} {\left\{v_{S}\right\}}_{t+\Delta t}^{i}={\left\{{\tilde{v}}_{S}\right\}}_{t+\Delta t}, \end{array}$$
  (A.2c) $$\begin{array}{c} {\left\{a_{S}\right\}}_{t+\Delta t}^{i}=\left\{0\right\}. \end{array}$$
(4) Set element counter, nel = 1.
(5) Set element iteration counter, *j* = 1.
  Subtract incremental displacement vector of external joints from global displacement vectors,
    (A.3) $$\begin{array}{c} {\left\{\Delta u_{E}\right\}}_{t+\Delta t}^{j}=\text{SUB}\left({\left\{\Delta u_{S}\right\}}_{t+\Delta t}^{i}\right). \end{array}$$
(6) Compute the element stiffness, mass, damping matrices, and element external and internal force vectors.
(7) Compute iterative and incremental displacement vectors of internal joints,
  (A.4a) {ΔuI}t+Δtj+1=[KII]t+Δtj(({ΔFI,gr}t+Δtj+1−{ΔFI,res}t+Δtj+1)      −[KIE]t+Δtj{ΔuE}t+Δti),
  (A.4b) $$\begin{array}{c} {\left\{u_{I}\right\}}_{t+\Delta t}^{j+1}={\left\{u_{I}\right\}}_{t+\Delta t}^{j}+{\left\{\Delta u_{I}\right\}}_{t+\Delta t}^{j+1}. \end{array}$$
(8) Check for convergence of iteration process ||{Δ*u*_*I*_}_*t*+Δ*t*_^*j*+1^|| (Euclidian norm of the unbalanced internal displacement vector at time step *t* + Δ*t* and element iteration *j*) using an element displacement tolerance (Tolelem).
  (a) If ||{Δ*u*_*I*_}_*t*+Δ*t*_^*j*+1^||/||∑_*m*=1_^*j*+1^{Δ*u*_*I*_}_*t*+Δ*t*_^*m*^|| ≤ Tolelem, convergence is achieved.
    Set
      (A.5a) $$\begin{array}{c} {\left\{u_{I}\right\}}_{t}={\left\{u_{I}\right\}}_{t+\Delta t}^{j+1},\;\;{\left\{v_{I}\right\}}_{t}={\left\{v_{I}\right\}}_{t+\Delta t}^{j+1},\;\;{\left\{a_{I}\right\}}_{t}={\left\{a_{I}\right\}}_{t+\Delta t}^{j+1}, \end{array}$$
      (A.5b) $$\begin{array}{c} {\left[K_{EE,F}\right]}_{t+\Delta t}^{j}={\left[K_{EE}\right]}_{t+\Delta t}^{j}-{\left[K_{EI}\right]}_{t+\Delta t}^{j}{\left({\left[K_{II}\right]}_{t+\Delta t}^{j}\right)}^{-1}{\left[K_{IE}\right]}_{t+\Delta t}^{j}, \end{array}$$
      (A.5c) $$\begin{array}{c} {\left\{F_{E,F}\right\}}_{t+\Delta t}^{i+1}={\left\{F_{E}\right\}}_{t+\Delta t}^{j+1}-{\left[K_{EI}\right]}_{t+\Delta t}^{j}{\left({\left[K_{II}\right]}_{t+\Delta t}^{j}\right)}^{-1}{\left\{F_{I}\right\}}_{t+\Delta t}^{j+1}, \end{array}$$
      (A.5d) [MEE,F]t+Δtj=[MEE]t+Δtj−[KEI]t+Δtj([KII]t+Δtj)−1[MIE]t+Δtj −[MEI]t+Δtj([KII]t+Δtj)−1[KIE]t+Δtj +[KEI]t+Δtj([KII]t+Δtj)−1 ×[MII]t+Δtj([KII]t+Δtj)−1[KIE]t+Δtj,
      (A.5e) [CEE,F]t+Δtj=[CEE]t+Δtj−[KEI]t+Δtj([KII]t+Δtj)−1[CIE]t+Δtj −[CEI]t+Δtj([KII]t+Δtj)−1[KIE]t+Δtj +[CEI]t+Δtj([KII]t+Δtj)−1[CII]t+Δtj ×([KII]t+Δtj)−1[KIE]t+Δtj,
      (A.5f) $$\begin{array}{c} \left[K_{S}\right]=\sum_{k=1}^{\text{nelem}}{\left[K_{EE,F}\right]}_{t+\Delta t}^{j},\;\;\left[C_{S}\right]=\sum_{k=1}^{\text{nelem}}{\left[C_{EE,F}\right]}_{t+\Delta t}^{j}, \end{array}$$
  (b) If global convergence is not achieved, set *j* = *j* + 1 and return to Step 5.
(9) Compute the effective dynamic stiffness matrix and the vector of unbalanced forces,
  (A.6a) $$\begin{array}{c} {\left[{\hat{K}}_{S}\right]}_{t+\Delta t}^{i}=\left(1-\alpha_{B}\right)A_{1}\left[M_{S}\right]+A_{2}{\left[C_{S}\right]}_{t+\Delta t}^{i}+{\left[K_{S}\right]}_{t+\Delta t}^{i}, \end{array}$$
  (A.6b) {ΔFS}t+Δti+1=(1−αB){FS}t+Δt+αB{FS,gr}t−{ΔFS,res}t+Δti+1 +{FS,stat}+[MS] ×(−(1−αB){aS}t+Δti+1+αB{aS}ti) −[CS]t+Δti{vS}t+Δti+1,
  where [*K*_*S*_]_*t*+Δ*t*_^*i*^ and {Δ*F*_*S*,res_}_*t*+Δ*t*_^*i*+1^ are the tangent stiffness matrix and the incremental restoring force vector of external joints on the global axis of the structure, respectively, which are assembled from the element contributions.
(10) Solve incremental displacements in the global axis,
  (A.7) $$\begin{array}{c} {\left[{\hat{K}}_{S}\right]}_{t+\Delta t}^{i}{\left\{\Delta u_{S}\right\}}_{t+\Delta t}^{i+1}={\left\{\Delta F_{S}\right\}}_{t+\Delta t}^{i+1}. \end{array}$$
(11) Update displacement, velocity, and acceleration vectors,
  (A.8a) $$\begin{array}{c} {\left\{u_{S}\right\}}_{t+\Delta t}^{i+1}={\left\{u_{S}\right\}}_{t+\Delta t}^{i}+{\left\{\Delta u_{S}\right\}}_{t+\Delta t}^{i+1}, \end{array}$$
  (A.8b) $$\begin{array}{c} {\left\{v_{S}\right\}}_{t+\Delta t}^{i+1}={\left\{{\tilde{v}}_{S}\right\}}_{t+\Delta t}+A_{2}\left({\left\{u_{S}\right\}}_{t+\Delta t}^{i+1}-{\left\{{\tilde{u}}_{S}\right\}}_{t+\Delta t}\right), \end{array}$$
  (A.8c) $$\begin{array}{c} {\left\{a_{S}\right\}}_{t+\Delta t}^{i+1}=A_{1}\left({\left\{u_{S}\right\}}_{t+\Delta t}^{i}-{\left\{{\tilde{u}}_{S}\right\}}_{t+\Delta t}\right). \end{array}$$
(12) Check for convergence of the iteration process ||{Δ*F*_*S*_}_*t*+Δ*t*_^*i*+1^|| (Euclidian norm of the unbalanced force vector at time step *t* + Δ*t* and global iteration *i*) using a force tolerance (Tolglo).
  (a) If ||{Δ*F*_*E*_}_*t*+Δ*t*_^*i*+1^||/||∑_*n*=1_^*i*+1^{Δ*F*_*E*_}_*t*+Δ*t*_^*n*^|| ≤ Tolglo, convergence is achieved.
    Set {*u*_*S*_}_*t*_ = {*u*_*S*_}_*t*+Δ*t*_^*i*+1^, {*v*_*S*_}_*t*_ = {*v*_*S*_}_*t*+Δ*t*_^*i*+1^, and {*a*_*S*_}_*t*_ = {*a*_*S*_}_*t*+Δ*t*_^*i*+1^.
  (b) If global convergence is not achieved, set *i* = *i* + 1 and return to Step 4.

## Abbreviations

FEA: Fibre element approach
FBEA: Fiber and Bernoulli-Euler approach
RC: Reinforced concrete.

### Symbols

*A*_*n*_: Area of the *n*th fiber/layer
{*a*_*S*_}: Acceleration vector of structure
*A*_Seg_: Cross-section area of segment
[*B*]: Strain-displacement matrix
[*C*_*EE*_]: Damping matrix for external freedoms
[*C*_*EE*,*F*_]: Frame damping matrix for external freedoms
[*C*_*EI*_]: Damping matrix for external-internal freedoms
[*C*_*IE*_]: Damping matrix for internal-external freedoms
[*C*_*II*_]: Damping matrix for internal freedoms
*E*_*O*,*n*_: Initial elasticity module of the *n*th fiber/layer
*E*_*T*,*n*_: Tangent elasticity module of the *n*th fiber/layer
{*F*_*E*_}: External load vector for external freedoms
{*F*_*I*_}: External load vector for internal freedoms
{*F*_*I*,gr_}: Dynamic external force vector for internal freedoms
{*F*_*I*,res_}: Dynamic restoring force vector for internal freedoms
{*F*_*S*,gr_}: Dynamic external force vector of structure
{*F*_*S*,res_}: Dynamic restoring force vector of structure
{*F*_*S*,stat_}: Static external force vector of structure
[*K*_*EE*_]: Stiffness matrix for external freedoms
[*K*_*EE*,*F*_]: Frame stiffness matrix for external freedoms
[*K*_*EI*_]: Stiffness matrix for external-internal freedoms
[*K*_*IE*_]: Stiffness matrix for internal-external freedoms
[*K*_*II*_]: Stiffness matrix for internal freedoms
[*K*_Seg_]: Stiffness matrix of the segment
*L*_Seg_: Length of segment
[*M*_*EE*_]: Mass matrix for external freedoms
[*M*_*EE*,*F*_]: Frame mass matrix for external freedoms
[*M*_*EI*_]: Mass matrix for external-internal freedoms
[*M*_*IE*_]: Mass matrix for internal-external freedoms
[*M*_*II*_]: Mass matrix for internal freedoms
[*M*_Seg_]: Mass matrix of the segment
*N*: Number of total fiber/layer on the fiber cross-section
[*N*(*ξ*)]: The element shape functions matrix
{*q*}: Displacement vector
*T*: Total kinetic energy of particle velocities on the cross-section of an element throughout its neutral axis
Tolelem: Tolerance value of element for Newton-Raphson procedure
Tolglo: Tolerance value of structure for Newton-Raphson procedure
*u*: Displacement in the axial directions of element local axis
{*u*_*E*_}: Displacement vector for external freedoms
{*u*_*I*_}: Displacement vector for internal freedoms
{*u*_*S*_}: Displacement vector of structure
$\left\{{\tilde{u}}_{S}\right\}$: Predicted displacement vector of structure
*v*: Displacement in the vertical directions of element local axis
{*v*_*S*_}: Velocity vector of structure
$\left\{{\tilde{v}}_{S}\right\}$: Predicted velocity vector of structure
*v*_*ξ*_: Particle velocity
*α*_*B*_: Bossak parameter
*α*_dam_: Rayleigh damping coefficient for mass matrix
*β*: First Newmark's coefficient
*β*_dam_: Rayleigh damping coefficient for stiffness matrix
*ε*_*xx*_: Strain of a point on a cross-section in the axial direction of element
*dε*_*ξ*,*n*_: Incremental axial strain of the *n*th fiber/layer in *ξ* local axis direction of a segment
*γ*: Second Newmark's coefficient
*η*_*n*_′: Vertical coordinates of the *n*th fiber/layer *η* in local axis direction of a segment
*ρ*: Mass density
Π: Total strain energy of a segment.
